# Timing and Motivations for Alternative Cancer Therapy With Insights From a Crowdfunding Platform: Cross-sectional Mixed Methods Study

**DOI:** 10.2196/34183

**Published:** 2022-06-07

**Authors:** John Peterson, Trevor Wilson, Joshua Gruhl, Sydney Davis, Jaxon Olsen, Matthew Parsons, Benjamin Kann, Angela Fagerlin, Melissa Watt, Skyler Johnson

**Affiliations:** 1 Department of Radiation Oncology Huntsman Cancer Institute University of Utah Salt Lake City, UT United States; 2 Department of Radiation Oncology Dana-Farber Cancer Institute and Brigham and Women's Hospital Harvard Medical School Boston, MA United States; 3 Department of Population Sciences University of Utah Salt Lake City, UT United States; 4 Veterans Affairs Salt Lake City Health Cancer System Salt Lake City, UT United States

**Keywords:** internet, health misinformation, online crowdfunding, alternative medicine, internet research ethics

## Abstract

**Background:**

Alternative cancer therapy is associated with increased mortality, but little is known about those who pursue it.

**Objective:**

We aimed to describe individuals’ motivations for using alternative cancer therapies and determine whether motivations differ based on individuals’ timing of seeking alternative therapies.

**Methods:**

We used data from 649 campaigns posted on the website GoFundMe between 2011 and 2019 for beneficiaries with cancer pursuing alternative therapy. The data were analyzed using a mixed methods approach. Campaigns were categorized by timing of alternative therapy (either before or after experiencing conventional therapy). Qualitative analysis identified motivational themes. Chi-square tests of independence and Fisher tests (all 2-sided) determined significant differences in the presence of motivational themes between groups.

**Results:**

The expression of concerns about the efficacy of conventional therapy was significantly more likely in campaigns for individuals who used conventional therapy first than in campaigns for individuals who started with alternative therapy (63.3% vs 41.7%; *P*<.001). Moreover, on comparing those who started with alternative therapy and those who switched from conventional to alternative therapy, those who started with alternative therapy more often expressed natural and holistic values (49.3% vs 27.0%; *P*<.001), expressed an unorthodox understanding of cancer (25.5% vs 16.4%; *P=.*004), referenced religious or spiritual beliefs (15.1% vs 8.9%; *P=.*01), perceived alternative treatment as efficacious (19.1% vs 10.2%; *P=.*001), and distrusted pharmaceutical companies (3.2% vs 0.5%; *P=*.04).

**Conclusions:**

Individuals sought treatments that reflected their values and beliefs, even if scientifically unfounded. Many individuals who reported prior conventional cancer therapy were motivated to pursue alternative treatments because they perceived the conventional treatments to be ineffective.

## Introduction

Alternative cancer therapy (ACT) is a subcategory of complementary and alternative medicine, a broad term defined by the National Cancer Institute to comprise the multitude of cancer treatment modalities outside the medical mainstream [1]. Among these treatment modalities are mind-body therapies, herbal supplements, special diets, and vitamin infusions. Complementary therapies are used alongside the standard of care as part of an integrative therapy plan created by a multidisciplinary care team, while ACT is used in place of the standard of care [1]. In other words, the same nonstandard treatment modality may be defined as either a complementary or an alternative therapy depending on whether it is applied as a complement to the standard of care (ie, complementary therapy) or as a replacement for it (ie, ACT) [2].

Research suggests that ACT use is common throughout the world and is seen by many patients as a curative form of cancer treatment [3-12]. Results from the 2018 American Society of Clinical Oncology Cancer Opinions Survey found that nearly 40% of adults surveyed in the United States believe that cancer can be cured through ACT alone [11]. These data are worrying because the efficacy of ACT for the treatment of cancer is either unproven or disproven and ACT use is linked to increased mortality among cancer patients who abandon conventional medical treatment [12]. Additionally, the high cost of ACT procedures and associated travel, surpassing US $50,000 per year for some cancer patients in the United States, may cause financial harm to patients and their families [13-15].

Studies on ACT use are challenging, since cancer patients may be hesitant to disclose ACT use to their providers [16]. This is particularly true in terms of studies on treatment decision making, since those who use ACT are often disconnected from standard medical systems that conduct qualitative research. Despite these challenges, research that improves the understanding of the complicated and multiphasic ways in which cancer patients decide to pursue ACT, including the timing and motivations for the decision, is necessary to improve health care providers’ ability to care for an already vulnerable patient population [17]. Online crowdfunding sites, such as GoFundMe, offer a novel approach for studying cancer patients’ treatment decisions [14,18,19]. Such sites are frequently used by cancer patients to raise money to pay for medical expenses, including both conventional and alternative therapies.

While previous studies have used data from crowdfunding sites to study individuals who use both classifications of unconventional therapy, we have focused our study on those individuals who state that they have chosen to pursue ACT exclusively [18,19]. This group is at the greatest risk for increased mortality and, therefore, warrants special attention [12]. The purposes of this study were to (1) describe individuals’ motivations for using ACT and (2) explore whether individuals who seek ACT before using the standard of care or conventional cancer therapy (CCT) differ from individuals who pursue ACT after using CCT. Addressing these questions will generate informative data that may help medical providers identify individuals likely to seek ACT, anticipate when they may be considering this decision, and proactively address potential motivations prior to an individual foregoing or abandoning CCT.

## Methods

### Design

This was a cross-sectional mixed methods study of GoFundMe campaigns created between 2011 and 2019 to raise money for ACT for individuals with cancer.

### Ethical Considerations

Special ethical concerns were considered as part of this internet-based research. This study did not involve an interaction or intervention with human subjects and was therefore exempt from institutional review board review [20]. Study data were extracted from publicly available campaigns in accordance with the GoFundMe terms of service, which states that “any information that is disclosed in [public campaigns] becomes public information for both us [GoFundMe] and other users to use and share” [21].

Despite the public nature of the data, we recognized the need to put additional protections in place, given the vulnerable position of individuals represented in the GoFundMe campaigns. Presenting qualitative data that were both publicly accessible and deeply personal created a unique challenge for the preservation of patient privacy, since any direct campaign quotes presented in this publication could be used to identify the organizers and beneficiaries, using internet search engine tools. Contacting each campaign organizer or beneficiary, some of whom were likely deceased, to obtain informed consent was not feasible. Considering these challenges and out of an abundance of caution, we opted to paraphrase the exemplary quotes presented in the qualitative results in a manner that removed identifiable characteristics (geography, age, cancer type, and gender, including replacing gender-specific pronouns with they/their/theirs) while retaining the sentiments and themes of the originals. Each paraphrased quote was queried by JP and TW using Google Search to ensure the campaigns could not be identified.

### Data Source and Selection

A custom web scraping code was developed and used to search GoFundMe for English language campaigns, using the term “alternative cancer” (Multimedia Appendix 1). The search was conducted on October 25, 2019, and yielded 795 campaigns that were initiated between 2011 and 2019. Each campaign was reviewed for eligibility according to the following criteria: written in English, inclusion of a campaign description, and raising of funds for an individual with cancer seeking ACT. To select campaigns that were seeking uniquely alternative rather than complementary therapies, the text of the campaigns was analyzed to determine whether the patients were using unconventional therapies simultaneously with conventional care or in place of conventional care. In cases where the patient had previously used CCT but had since stopped, the campaign was classified as ACT. If the described therapy was complementary or if the intent was ambiguous, the campaign was omitted. If a campaign was found to be a duplicate of another, only 1 version was included in the study. Among the 795 campaigns, 17 did not meet these criteria and were excluded. The remaining 778 campaigns were reviewed to determine the timing of the beneficiary’s decision to pursue ACT. A campaign was only included if it could be determined that the beneficiary sought ACT either before or after experiencing CCT. Among the 778 campaigns, 129 did not contain sufficient details to determine timing and were excluded, leaving 649 campaigns in the final analysis.

### Statistical Analysis

Clinical, demographic, and treatment data were extracted for the 649 campaigns. Variables included the individual’s gender, nation of residence, primary cancer type, cancer stage, and ACT modality. Each campaign was categorized by the timing of the individual’s decision to pursue ACT. “ACT first” included individuals who had started their treatment with ACT, and “ACT after CCT” included individuals who had used CCT prior to seeking ACT. Campaigns were categorized into the “ACT after CCT” group if the patients had ever received CCT prior to seeking ACT, including for an earlier occurrence of the same cancer or a different cancer. To categorize cases for timing, 50 cases were dually classified by 2 independent coders (κ=0.750), discordant cases were discussed, and procedures were clarified before commencing independent classification of the remaining campaigns.

The text of the campaign description was analyzed in ATLAS.ti 9 using applied thematic analysis techniques [22]. First, a subset of 100 cases was inductively coded by 2 independent analysts to identify themes related to motivations for using ACTs. Themes were considered “motivational” if they initiated, guided, or informed the decisions of beneficiaries to pursue ACT. They may not have been the sole rationale, but they were prominent enough that the beneficiaries felt they were important to include in their calls for donations. The codes were discussed in the larger research team, and consensus was reached on a set of codes that best captured themes across the campaigns. A codebook was developed that included parent and child codes, with definitions and exemplary quotes. The 2 analysts then dually coded 50 transcripts (Krippendorff α=.745). Discordant codes were discussed for consensus, and modifications were made to the codebook to clarify code definitions. The remaining campaigns were thereafter coded individually by the 2 analysts. Code reports were generated to synthesize the text associated with each code and to quantify the number of campaigns in which each code appeared.

Statistical analyses were performed using Stata, version 16.1 (Stata Corp). The demographic, clinical, and treatment characteristics of the campaigns were described, and associations with treatment timing (ACT first vs ACT after CCT) were examined using chi-square tests of independence (for variables with frequencies ≥5) and Fisher exact tests (for variables with frequencies <5). Each code representing a motivational theme was transformed into a variable, and campaigns were assigned as having that theme present in the text or not. Chi-square tests of independence or Fisher exact tests were used to assess whether the presence of each motivational theme was associated with treatment timing. All statistical tests were 2-sided.

## Results

### Demographic and Clinical Characteristics of the Sample

The demographic and clinical characteristics of the individuals represented by the 649 campaigns are shown in Table 1. Details about the ACT modalities individuals pursued are shown in Table 2. Of the 649 individuals represented by the campaigns, 371 (57.2%) sought ACT after using CCT and 278 (42.8%) pursued ACT first.

**Table 1:** Demographics and clinical breakdown of the sample by timing of the decision to use alternative cancer therapy.

Variable			Total campaigns (N=649), n (%)		ACT^a^ first (N=278), n (%)		ACT after CCT^b^ (N=371), n (%)	*P* value^c^
**Gender**								.47
	Female	417 (64.3)		183 (65.8)		234 (63.1)		
	Male	232 (35.7)		95 (34.2)		137 (36.9)		
**Cancer type**								<.001
	Breast	171 (26.3)		82 (29.5)		89 (24.0)		
	Colorectal	70 (10.8)		24 (8.6)		46 (12.6)		
	Lung	36 (5.5)		15 (5.4)		21 (5.7)		
	Head and neck	35 (5.4)		25 (9.0)		10 (2.7)		
	Brain	30 (4.6)		8 (2.9)		22 (5.9)		
	Esophagus/gastric	28 (4.3)		16 (5.8)		12 (3.2)		
	Ovarian	28 (4.3)		7 (2.5)		21 (5.7)		
	Pancreas	26 (4.0)		5 (1.8)		21 (5.7)		
	Bone and soft tissue	24 (3.7)		9 (3.2)		15 (4.0)		
	Lymphoma	22 (3.4)		11 (4.0)		11 (3.0)		
	Other^d^	179 (27.6)		76 (27.3)		103 (27.8)		
**Cancer stage^e^**								.03
	I, II, or III	76 (20.8)		38 (26.6)		38 (17.1)		
	IV	289 (79.2)		105 (73.4)		184 (82.9)		
**Primary residence**								.06
	United States	524 (80.7)		235 (84.5)		289 (77.9)		
	Europe	57 (8.8)		23 (8.3)		34 (9.2)		
	Canada	43 (6.6)		15 (5.4)		28 (7.5)		
	Other	25 (3.9)		5 (1.8)		20 (5.4)		

^a^ACT: alternative cancer therapy.

^b^CCT: conventional cancer therapy.

^c^From chi-square tests comparing patients in the “ACT first” and “ACT after CCT” groups.

^d^Other cancers include anal, cervix, endometrial, leukemia, melanoma, nonmelanoma skin, liver and biliary, kidney, multiple myeloma, prostate, bladder, neuroendocrine, thyroid, testicular, vulvar, and unspecified.

^e^Cancer stage was reported in 365 campaigns, with 143 in the “ACT first” group and 222 in the “ACT after CCT” group. These numbers were used as the denominators for each cancer stage timing category.

**Table 2:** Details of alternative cancer therapies pursued by the timing of the decision to use alternative cancer therapy.

Proposed ACT^a^	Total campaigns (N=649), n (%)	ACT first (N=278), n (%)	ACT after CCT^b^ (N=371), n (%)	*P* value
Special diets	187 (28.8)	92 (33.1)	95 (25.6)	.04
Vitamins and minerals	155 (23.9)	77 (27.7)	78 (21.0)	.05
Supplements	128 (19.7)	70 (25.2)	58 (15.6)	.003
Intravenous infusions	119 (18.3)	55 (19.8)	64 (17.3)	.41
Herbs and botanicals	101 (15.6)	53 (19.1)	48 (12.9)	.03
Heat/light/sauna	65 (10.0)	39 (14.0)	26 (7.0)	.003
Oxygen therapy (hyperbaric, etc)	62 (9.6)	32 (11.5)	30 (8.1)	.14
Unknown injections	51 (7.9)	29 (10.4)	22 (5.9)	.04
Homeopathy and naturopathy	44 (6.8)	20 (7.2)	24 (6.5)	.72
Ozone therapy (topical, intravenous, intramuscular)	40 (6.2)	20 (7.2)	20 (5.4)	.34
Enemas	35 (5.4)	14 (5.0)	21 (5.7)	.73
Prayer	32 (4.9)	17 (6.1)	15 (4.0)	.23
Yoga or exercise	30 (4.6)	15 (5.4)	15 (4.0)	.42
Insulin potentiation therapy	27 (4.2)	17 (6.1)	10 (2.7)	.03
Electromagnetic therapies^c^	25 (3.9)	11 (4.0)	14 (3.8)	.90
Massage	17 (2.6)	9 (3.2)	8 (2.2)	.39
Acupuncture	14 (2.2)	8 (2.9)	6 (1.6)	.27
Meditation	14 (2.2)	6 (2.2)	8 (2.2)	>.99
Other	19 (2.9)	14 (5.0)	5 (1.3)	.006

^a^ACT: alternative cancer therapy.

^b^CCT: conventional cancer therapy.

^c^Includes pulsed electromagnetic frequency therapy, Rife, electrocancer therapy, and galvanotherapy.

### Motivational Themes

On examining the stated motivations for pursuing ACT, 4 primary themes (“Dissatisfaction with CCT,” “Compatibility with belief system,” “Desire for greater personal control,” and “Perceived efficacy of ACT”) were identified. Subthemes emerged under “Dissatisfaction with CCT” (“Perceived inefficacy,” “Adverse effects,” and “Financial concerns”) and “Compatibility with belief system” (“Natural and holistic values,” “Unorthodox understanding of cancer and/or therapy,” “Distrust of medical professionals and hospitals,” “Religious/spiritual reasons,” and “Distrust of pharmaceutical companies”). The 4 most common motivational themes were “Perceived inefficacy” of CCT (n=351, 54.1%), “Adverse effects” of CCT (n=281, 43.3%), “Natural and holistic values” (n=237, 36.5%), and “Unorthodox understanding of cancer and/or therapy” (n=132, 20.3%). Multimedia Appendix 2 provides a summary of all themes and subthemes, exemplary quotations, and frequencies of campaigns in which these themes occurred.

### Comparison Between the Timing Groups

Most cancer types seen in this study were found to be significantly more represented among campaigns for beneficiaries who were seeking ACT after CCT (*P<*.001) (Table 1). Only beneficiaries reporting head and neck cancers or esophageal/gastric cancers were more represented among campaigns that sought ACT first (9.0% vs 2.7% and 5.8% vs 3.2%, respectively; *P<.*001). All other cancers (breast, colorectal, lung, brain, ovarian, pancreatic, bone and soft tissue, and lymphoma) were more common among campaigns that reported seeking ACT after CCT (*P<.*001) (Table 1). Cancer stage was reported in 365 (56.2%) campaigns analyzed. Among these campaigns, stage IV cancers were significantly more common in individuals who were seeking ACT after CCT than ACT first (82.9% vs 73.4%; *P=*.03), while those reporting stage I, II, and III cancers were more often seeking ACT first than ACT after CCT (26.6% vs 17.1%; *P=.*03) (Table 1).

The campaigns for beneficiaries who pursued ACT as first-line treatment were significantly more likely to seek the following 8 of 19 classes of ACT modalities identified in this study (Table 2): special diets (33.1% vs 25.6%; *P=*.04); vitamins and minerals (27.7% vs 21.0%; *P=.*05); supplements (25.2% vs 15.6%; *P=.*003); herbs and botanicals (19.1% vs 12.9%; *P=.*03); heat, light, and sauna therapies (14.0% vs 7.0%; *P=.*003); unknown injections (10.4% vs 5.9%; *P=.*04); insulin potentiation therapy (6.1% vs 2.7%; *P=.*03); and other therapies, including electrical therapies such as galvanotherapy and Rife therapy (5.0% vs 1.3%; *P=.*006).

Campaigns for individuals who started with ACT were significantly more likely to express natural and holistic values (49.3% vs 27.0%; *P*<.001), demonstrate an unorthodox understanding of cancer or cancer treatment (25.5% vs 16.4%; *P=.*004), cite their religious or spiritual beliefs (15.1% vs 8.9%; *P=.*01), mention distrust of pharmaceutical companies (3.2% vs 0.5%; *P=.*01), and make claims about the efficacy of the chosen ACT (19.1% vs 10.2%; *P=.*001) (Multimedia Appendix 2). Campaigns for individuals who pursued ACT after CCT were significantly more likely to perceive CCT to be ineffective (63.3% vs 41.7%; *P*<.001) (Multimedia Appendix 2).

## Discussion

### Principal Findings

This study highlights the diversity of motivations for choosing to pursue ACT present among a small sample of English-speaking GoFundMe users. Most individuals featured in the GoFundMe campaigns had prior experience with CCT and pursued ACT primarily because of their perception that CCT was not effective. Not surprisingly, metastatic disease and concerns about the inefficacy of CCT were both significantly more common among the same class of campaigns. The limited treatment options available to these individuals may have prompted an interest in ACT as a last resort. This may seem acceptable to maintain hope and preserve patient autonomy; however, improved communication between physicians and patients is needed to discuss the physical and financial risks of unproven treatments. When patients have exhausted all options for evidence-based therapies, shared decision-making and coordination with supportive oncology services, palliative care, and other necessary providers should be prioritized and initiated early in their care to improve their care experience and maintain quality of life.

The role that beliefs and values play in guiding the decisions regarding cancer care can be seen in the campaigns for individuals in our sample, who chose to pursue ACT as their first mode of treatment. Campaigns for these individuals were more likely to express a desire for care that was consistent with their personal beliefs, particularly a value-based preference for natural healing. In some campaigns the desire for a more “natural” therapy was closely tied to an incorrect understanding of cancer biology, often surrounding the immune system’s capacity to fight cancer. A total of 132 (20.3%) campaigns cited pseudoscientific information as the reason for pursuing ACT, underscoring the impact of medical misinformation, often from online sources, in persuading individuals to use cancer treatments that are not evidence-based [23]. Frequently, seeking natural care was conveyed as a mark of faith. Rather than putting their confidence in secular science, the beneficiaries stated that they were manifesting their trust in God’s ability to heal by refusing CCT. The beliefs that motivated individuals to use ACT as a first-line therapy commonly drew from multiple sources, blending in a way that was deeply personal and grounded in one’s identity and core values.

These results highlight the dilemma faced by medical providers who strive to respect patient autonomy while encouraging patients to pursue evidence-based treatment [24]. Despite these challenges, medical communication research offers some guidance on facilitating open goal-concordant and patient-centered care conversations in these situations. A medical provider’s ability to actively listen, express compassion, and build a relationship with his or her patients has been shown to increase trust and counter false medical information [2,25]. Establishing a strong therapeutic alliance may make it easier for patients who lack trust in mainstream medicine or hold unorthodox medical beliefs to discuss their concerns more freely with medical providers.

It remains unclear why certain ACT modalities, such as supplements and herbal remedies, were more represented among campaigns seeking ACT first. It is possible that this is a product of the selection bias generated by gathering information from English-speaking individuals, most of whom are US residents with internet access. A larger more diverse sample might have produced different results. The relative frequency of these modalities is similar to the findings of previous research using GoFundMe data. In their 2018 investigation into complementary and alternative cancer treatment use, Song et al found special diets, herbal remedies, oral vitamin and mineral supplements, and vitamin injections to be among the top 10 most frequently used modalities [19]. The results of this paper, while limited to alternative cancer treatments, nonetheless also found special diets, oral vitamins and minerals, supplements, herbal remedies, and intravenous vitamins to be the most sought-after forms of ACT. An additional study is warranted to understand why these therapies are consistently desirable. Furthermore, as research expands the number of evidence-based options available to patients, the list of therapies that are considered CCTs will continue to evolve. It may be valuable to monitor how emerging therapeutic approaches, including immunotherapy, nanostructures, and tumor-selective delivery of chemotherapy, will impact trends in the use of ACT modalities [26,27].

### Internet Research Ethics

In conducting this study, we carefully weighed the ethical considerations of using social media data and took steps to protect the identity of vulnerable individuals who created GoFundMe campaigns. In recent years, internet research has highlighted the importance of protecting the agency and privacy of online research subjects [28-30]. The descriptive approach taken in this study yielded insights into medical decision-making without interacting or providing an intervention with human subjects [20]. Multimedia Appendix 2 provides examples of how public, but potentially sensitive, qualitative data can be presented in a way that preserves both its meaning and the sources’ privacy [29].

### Limitations

This novel approach to conducting research with a particularly difficult-to-study cancer population provides important descriptive information about individuals who pursue ACT. Nevertheless, the study’s findings must be considered in light of its limitations, primarily the reliance on campaign texts that were written for the purpose of soliciting financial support for individuals seeking ACT. Here, we attempted to identify motivational themes among those using only alternative therapies. The term “alternative cancer” was used to potentially exclude those patients who are using reiki, homeopathy, Gerson therapy, or other specific therapies with their CCTs, or are otherwise defined as complementary medicine users. We acknowledge that in an effort to exclude those receiving complementary medicines, we may have underascertained some users of ACTs. However, this term is highly specific and still resulted in the largest qualitative study, known to date, of patients receiving alternative medicines for cancer. Information content was inconsistent across campaigns, and some campaign descriptions were written by close friends and family rather than directly by the individuals living with cancer. We felt that it was still appropriate to include these campaigns authored by close friends and family because they were often intimately involved throughout the diagnosis and medical decision-making process. Additionally, we did not want to exclude campaigns for patients whose health condition or technological literacy may have prevented them from independently writing and organizing their GoFundMe campaigns. Moreover, we acknowledge the possibility that fraudulent campaigns were inadvertently included in this study and acknowledge our dependence on the GoFundMe fraud detection system to minimize this risk. To mitigate the possibility of including fraudulent campaigns, we excluded campaigns that did not include text or information detailed enough to determine the timing of alternative therapy [21]. Some characteristics of the data may limit their generalizability. Not all ACT users utilize crowdfunding, and our data likely excluded individuals with limited access to the internet, individuals without broad social networks, and individuals who could afford to self-fund their treatments. Though the sample size of 649 campaigns is in line with other studies performed using GoFundMe data, the inclusion criterion of English language presents a bias toward an English-speaking US-based population, which limits the generalizability of these results to a broader population. Finally, the individuals represented by the campaigns were not contacted, and thus, the information provided about their treatment and diagnosis was not verifiable. Notwithstanding these issues, this research yielded a large amount of data that could serve as a starting place for future important investigations into larger and more generalizable populations.

### Conclusions

Individuals represented by the GoFundMe campaigns in our sample chose to pursue ACT at different points in time during their treatment course, and the sequence of their decisions is associated with specific clinical profiles and motivations. The results of this study emphasize the importance of providers having candid and compassionate discussions with their patients throughout the course of treatment, starting at diagnosis and continuing as the disease and treatment progress. Just as individuals’ motivations differed depending on when they chose to pursue ACT, so too should providers’ responses. By learning what makes ACT an attractive option, medical providers can better respond to patients’ beliefs and values, and advocate for evidence-based treatment.

## Appendix

Multimedia Appendix 1 Web scraping code.

Multimedia Appendix 2 Description of key elements of patients’ reasons for pursuing alternative cancer therapy either before or after using conventional cancer therapy.

## Abbreviations

ACT: alternative cancer therapy
CCT: conventional cancer therapy
