# Supplementary material for: Timing and Motivations for Alternative Cancer Therapy With Insights From a Crowdfunding Platform: Cross-sectional Mixed Methods Study
Source: JMIR Cancer. 2022 Jun 7;8(2):e34183. doi: 10.2196/34183 (PMC9214612; doi:10.2196/34183)
Supplement: Multimedia Appendix 2 [file cancer_v8i2e34183_app2.docx]

**Multimedia Appendix 2.** Description of key elements of patients’ reasons for pursuing alternative cancer therapy either before or after using conventional cancer therapy.

| Theme | Sub-theme^a^ | Summary | Paraphrased Quotations^c^ | No. of campaigns (%) n = 649 | ACT first (%) n = 278 | ACT after CCT (%) n = 371 | *P*-value^b^ |
| --- | --- | --- | --- | --- | --- | --- | --- |
|  |  |  |  |  |  |  |  |
| Dissatisfaction with CCT | Perceived inefficacy | An expression of doubt that beginning CCT will effectively treat cancer or that continuing CCT will have any meaningful benefit. | The beneficiary has undergone numerous conventional therapies, but their cancer has continued to spread. | 351 (54.1) | 116 (41.7) | 235 (63.3) | <.0001 |
|  |  |  | Because conventional therapy has been unsuccessful, the beneficiary has to seek alternative cancer treatment even though it was not covered by insurance. |  |  |  |  |
|  | Adverse effects | An expressed concern about CCT's adverse effects, or a desire to avoid or stop experiencing them. This includes adverse effects that have already been experienced or potential adverse effects that the author believes could occur. | The beneficiary believes that chemotherapy will be too much for their body to handle and will not improve their quality of life. | 281 (43.3) | 110 (39.6) | 171 (46.1) | .10 |
|  |  |  | Undergoing conventional cancer therapy only left the beneficiary with more problems than they had before treatment. |  |  |  |  |
|  | Financial concerns | An expressed concern that pursuing ACT therapy is a better financial decision than undergoing CCT. | The beneficiary is very opposed to conventional therapy because they estimate the cost would be higher than if they used alternative therapy. | 6 (0.9) | 4 (1.4) | 2 (0.5) | .41 |
|  |  |  | The beneficiary is optimistic that pursuing alternative therapy rather than conventional therapy will liberate them from further financial entanglements and medical expenses. |  |  |  |  |
| Compatibility with belief system | Natural and Holistic values | An expressed confidence in the power of "natural healing" and/or confidence in the "natural" aspects of alternative medicine; also, an expressed belief that alternative therapies treat health issues on multiple levels—either by simultaneously treating health problems unrelated to cancer or by addressing and larger underlying problem of which cancer is a symptom. | After deciding to stop treating their cancer with what they consider to be artificial medicines, the beneficiary is now raising money to travel to clinics where they can learn about more natural forms of healing that they believe will cure their cancer through the fostering of mind, body and spirit. | 237 (36.5) | 137 (49.3) | 100 (27.0) | <.0001 |
|  |  |  | The beneficiary has decided to stop chemotherapy and instead treat their cancer with supplements, herbal remedies, and dietary changes which they feel work more harmoniously with the body. |  |  |  |  |
|  | Unorthodox understanding of cancer and/or therapy | An explanation for the patient's cancer that is not based in accepted medical science or an exaggerated claim about the mechanisms of alternative treatments. These often reference the immune system—either misunderstanding its role in cancer or overemphasizing its importance in cancer treatment. | The beneficiary believes their cancer arose because of years of chronic stress and emotional trauma that suppressed their immune system so it could not carry out its necessary cancer-fighting role. | 132 (20.3) | 71 (25.5) | 61 (16.4) | .004 |
|  |  |  | The beneficiary is fundraising for an alternative therapy that uses naturopathy and an alternative form of chemotherapy that they believe will drastically reduce the side effects of conventional chemotherapy, while at the same time building the immune system. |  |  |  |  |
|  | Distrust of medical professionals and hospitals | An expressed lack of confidence in medical providers due to personal conspiratorial views, experiencing a provider's a poor bedside manner, perceived medical error, or malpractice. | The beneficiary expresses suspicion about the way in which doctors quickly steer patients toward conventional cancer therapies while not discussing alternative therapy. They boldly state that their mission is to expose the truth and show the real effectiveness of alternative therapies. | 84 (12.9) | 39 (14.0) | 45 (12.1) | .48 |
|  |  |  | The beneficiary reports a traumatic experience with doctors and hospitals. They say that they were showing signs of cancer for a long time but treatment was delayed by repeated misdiagnosis, harsh testing, and no clear answers which led to mistrust and confusion. |  |  |  |  |
|  | Religious/spiritual reasons | The patient’s religious or spiritual beliefs, including specific spiritual or supernatural experiences, have influenced their decision to seek alternative over mainstream therapy. | The beneficiary believes that the human body was designed by God with the ability to heal from cancer without conventional therapy. They go on to say that they feel God is telling them to pursue an alternative mode of cancer treatment. | 75 (11.6) | 42 (15.1) | 33 (8.9) | .01 |
|  |  |  | After having a profound spiritual experience, in which they made a fervent prayer to God asking to survive their cancer, the beneficiary when into remission. The cancer has since returned and is now more aggressive, but the beneficiary's belief in God motivated them to search out a clinic that uses alternative therapies. |  |  |  |  |
|  | Distrust of pharmaceutical companies | A critical of conspiratorial statement about the pharmaceutical industry that in turn questions the merit of using CCT. | The beneficiary expresses disdain for the agenda of big pharmaceutical companies, especially for the high cost of cancer drug prescriptions. | 11 (1.7) | 9 (3.2) | 2 (0.5) | .01 |
|  |  |  | After receiving chemoradiation, the beneficiary opted to switch and try treatments that they believe are have shown success but are not widely marketed because they are not profitable for pharmaceutical companies. |  |  |  |  |
| Desire for greater personal control | —^c^ | A claim that using ACT therapy will afford a greater sense of freedom, self-respect, empowerment, and control over their disease. | After considering many different treatment options, the beneficiary decided to get treatment from a prominent alternative therapy provider who had created a custom treatment plan based on their individual body's chemistry in order to ensure the best possible outcome. | 105 (16.2) | 48 (17.3) | 57 (15.4) | .51 |
|  |  |  | The beneficiary refused to hear that they would die or be incapable of healing. Pursuing alternative therapy gave them a sense of relief that they no longer had to submit to or rely on the will of others for their healing. |  |  |  |  |
| Perceived efficacy of ACT | — ^c^ | A description of the perceived success of ACT treatment including decreased pain, improved mobility, favorable imaging and test results. Whether or not the improvements are clinically significant or objectively true does not matter so much the patient's belief in them. | After their first round of alternative therapy, the beneficiary quickly experienced physical relief and is excited for the coming benefits of receiving the treatments on a regular basis. | 91 (14.0) | 53 (19.1) | 38 (10.2) | .001 |
|  |  |  | When the beneficiary first arrived at the alternative therapy clinic they needed to use a wheelchair to get to most of their meals. They are now using their walker independently and can even stand without aid for short periods of time. |  |  |  |  |
